# In Pursuit of Transformational Clinical Solutions

**DOI:** 10.3390/jcm13113021

**Published:** 2024-05-21

**Authors:** Kent Doi

**Affiliations:** Department of Emergency and Critical Care Medicine, University of Tokyo, Tokyo 113-8655, Japan; kentdoi@m.u-tokyo.ac.jp

It gives me great pleasure to introduce myself as the new Co-Editor-in-Chief of the *Journal of Clinical Medicine* (JCM). 

I bring to the role many years of experience, as a clinician and editor, and, perhaps even more than this, an unwavering commitment to pushing the boundaries of our knowledge and capabilities in clinical medicine. 

Journals, such as JCM, play a key role in disseminating new research findings, encouraging scholarly debate, and helping the medical community to develop innovative new clinical approaches. It is my hope that, in my new role, I can build on the excellent work that has brought JCM to where it is today and help to make it even more effective in addressing currently unmet needs for clinicians and patients alike.

JCM is an international, peer-reviewed, and open access journal of clinical medicine, publishing 24 issues online per year. 

As an open access publication, JCM is free for readers, and its costs are covered by the article processing charges (APCs) provided by the individual authors or their institutions. Indexed within Scopus, SCIE (Web of Science), PubMed, PMC, Embase, CAPlus/SciFinder, and other databases, JCM enjoys high visibility, with a Journal Rank of JCR Q2 (Medicine, General & Internal) and a CiteScore of Q1 (General Medicine). For contributors, the journal offers a prompt, robust, and transparent peer-review and editorial process, facilitating the rapid dissemination and discussion of the latest research in our field.

I am very happy to be part of this editorial approach, as I believe it best serves the needs of clinical medicine today. Founded in 2012, JCM can look back on 12 years of valuable activity. 

I have taken on this new task in order to bring the concept of JCM closer to my colleagues—especially in Japan. The current rejection rate of 62% makes it possible to quickly and efficiently publish well-written scientific papers. This is important because the more data can be shared quickly, the more we can all benefit.

I am also pleased to see that MDPI offers generous discounts on APCs for institutions interested in centralized payment. MDPI calls this the International Open Access Program (IOAP): https://www.mdpi.com/ioap (16 May 2024). 

It is my belief that in a field as complex as clinical medicine today, success is predicated on pursuing simplicity without falling into the simplistic. JCM’s editorial approach is one example of that simplicity. 

I hope that readers will value this, and that potential contributors and reviewers will be encouraged to submit their work and help move us forward in our pursuit of transformational clinical solutions for the patient communities we serve. On a personal note, I hope that many of my international colleagues—especially my colleagues in Japan—will wish to join us in this endeavor.

This year, I will attend the Annual Meeting of the Japanese Society for Dialysis Therapy (JSDT), and I will be available for a ‘Meet-the-Editor’ meeting at the MDPI booth at the exhibition area. Please do not hesitate to join us from 7 to 9 June 2024 in Yokohama, Japan.

